# Synergistic Effects of Graphene/Carbon Nanotubes Hybrid Coating on the Interfacial and Mechanical Properties of Fiber Composites

**DOI:** 10.3390/ma13061457

**Published:** 2020-03-23

**Authors:** Wenzhen Qin, Chao Chen, Jianping Zhou, Jiangyan Meng

**Affiliations:** 1 School of Materials Science and Engineering, Nanchang Hangkong University, Nanchang 330063, China; 18720086617@163.com (C.C.); zhoujp@nchu.edu.cn (J.Z.); mengjiangyan@nchu.edu.cn (J.M.); 2 Jiangxi Provincial Engineering Research Center for Surface Technology of Aeronautical Materials, Nanchang Hangkong University, Nanchang 330063, China

**Keywords:** graphene/carbon nanotube hybrid coating, nano-structure, mechanical properties, carbon fiber composites

## Abstract

In this study, the graphene nanoplates (GnPs) and carbon nanotubes (CNTs) are simultaneous deposited on carbon fiber (CF) surface by fiber sizing method. The synergistic effect between GnPs and CNTs in increasing the interfacial and mechanical properties of carbon fiber reinforcement epoxy composites (CFRP) is investigated. The fracture surfaces of the CFRP composites indicated that GnPs/CNTs hybrid coating exhibited the best interfacial and mechanical performance in all coating sample. The interlaminar shear strength of GnPs/CNTs hybrid coated CFRP composites was 90% higher than non-coated CF composites. The flexural and tensile strength of CFRP composites with GnPs/CNTs hybrid coating have an improvement of 52% and 70%, respectively, compared to non-coated CF.

## 1. Introduction

Owing to their excellent mechanical properties, high specific strength, good electrical properties and outstanding design flexibility, carbon fiber reinforced epoxy composites have attracted much attention and been widely used in aerospace, automotive and sports industries [1,2,3,4,5]. The ultimate performance of the composites largely affects by the interface properties between carbon fiber (CF) and matrix [6]. It is now well established that a designed interface can improve the structural integrity of composites and ensure the efficient stress transfer from the matrix to the reinforcement fiber [7,8,9]. However, the interfacial interactions between CF and matrix tends to be weak due to the hydrophobic and relatively low functionality of CF surface, which impede the advantages of the CF and affect the mechanical properties of its composites [10,11]. Therefore, extensive research studies have been focused on the surface treatment of CF using various methods, which could improve the interfacial interaction between CF and matrix. Previous treatment methods include fiber sizing [12], electrochemical oxidation [13], grafting [14], plasma treatment [15] and microwave etching [16]. Fiber sizing plays a major role in modification of CF surface and has advantages for large scale manufacturing which is often used in the CF production process [17,18]. 

In recent years, the introduction of nanoparticles into the CF reinforcement composites has been attracted great interest due to the excellent mechanical properties and reinforcement potential of nanoparticles. Further, simulation studies have found that the CF composites with nanoparticles can reduce stress concentrations the interface sand improve their mechanical properties [19,20]. It has been reported that the incorporation of graphene nanoplates [21] and carbon nanotubes [22] in the sizing could enhance the interface and mechanical properties of carbon fiber reinforcement epoxy composites (CFRP) composites. 

One dimensional (1D) carbon nanotubes (CNTs) is high surface area, high strength and flexibility. CNTs is a valuable candidate to improve the interface properties and manufacture the multifunctional carbon fiber reinforcement composite since CNTs has excellent mechanical, electrical and thermal properties [23,24]. Research studies have shown improvements of interfacial and mechanical properties in carbon fiber reinforce composites by adding CNTs in sizing solution, due to increase mechanical interlocking and chemical bonding between CF and matrix [25]. Li et al. [26] applied CNTs sizing on the CF surface by an aqueous suspension deposition method. The CNTs healed CF surface flaws and were the bridges between CF and matrix to reduce the stress concentrations. Yao et al. [27] reported that interlaminar shear strength and flexural strength of CNTs coated CFRP composites increased to 13% and 20% compared to non-coated CFRP composites. It was indicated that gradient interfacial structure with various thick- nesses was formed due to CNT incorporation at the CF/EP interphase. However, CNTs tends to aggregate due to their large aspect ratio and strong Van der Waals forces, that result in difficult dispersion in sizing solution and consequently unevenly coated on the surface of CF [28]. 

Graphene nanoplates (GnPs), a monolayer of sp2-hybridized carbon atoms organized in a two-dimensional (2D) lattice, have attracted considerable attention in recently years due to its large specific surface area, excellent mechanical properties and electric properties [29]. Some researchers have employed 2D GnPs to modify CF and CF/epoxy composites. Chen et al. [7] prepared graphene oxide and polyether imide (PEI) complex sizing, and then the complex sizing was coated on CF surface. The CF/PEEK composites with graphene oxide plus PEI complex sizing have an enhancement of interfacial shear strength. The graphene oxide sheet can block the initial cracks and reduce the stress intensity factors at the crack tips [30]. Recently our group has [21,31] used GnPs/epoxy sizing as a function coating to modify the surface of CF. The interfacial adhesion, mechanical properties and electrical properties of GnPs modified CF/epoxy composites were improved. However, GnPs presents the dispersion problem as well as CNTs, because the large surface area of GnPs causes in strong van der Waals forces and π-π inter-planer stacking [32]. Consequently, the GnPs and CNTs are unevenly deposited on CF surface, so that the properties of GnPs and CNTs coated CFRP composites reduce significantly compared with non-coated CFRP composites [33,34]. In order to solve the problem, the GnPs and CNTs were usually acid oxidized that can increase the dispersion of GnPs and CNTs in sizing solvent. However, the reaction conditions of oxidations are severe, complex and difficult to control [35]. Furthermore, the graphitic structure and the intrinsic properties of GnPs and CNTs may be serious destroyed by oxidation [36]. Herein, it depends on obtaining uniform dispersion of GnPs and CNTs in the sizing solvent, if researchers want to successfully achieve the multifunctional CFRP composites. As the more recently reported, the addition of different geometric shapes nanoparticles could be an effective way to achieve homogeneous dispersion [37,38]. The composites modified by hybrids nanoparticles have achieved better interfacial, mechanical, electrical and thermal properties [39]. It was reported that the long and flexible 1D nanoparticles bridged adjacent 2D nanoparticles and inhibited their aggregation, leading to an improvement of contact surface area between hybrid nanoparticles structures and the polymer [40]. A synergetic effect in terms of fracture energy and fatigue [37], tensile strength and elastic modulus [41] were reported in carbon nanofibers (CNF) and GnP hybrid nanoparticles composites. Better mechanical and thermal properties were reported in epoxy composites with 0.9 wt.% multi-graphene platelets (MGP) and 0.1 wt.% CNTs due to a more efficient network formation [37]. However, most research studies have reported that hybrid nanoparticles were used to modify epoxy as mentioned above, utilizing hybrid coating as a functional sizing agent on CF surfaces has been less investigated.

Inspired by the work and thought mentioned above, in this work, GnPs/CNTs hybrid functional coating were prepared and coated on carbon fiber surfaces by fiber sizing. GnPs/CNTs hybrid-coated carbon fiber/epoxy laminated composites were manufactured by hot press molding. The microstructures of the GnPs/CNTs hybrid-coated carbon fiber were observed by scanning electron microscopy (SEM). The mechanical properties of GnPs/CNTs hybrid-coated carbon fiber/epoxy composites were tested. The flexural strength fracture morphology was analyzed, and the mechanisms of interface strengthening were discussed. 

## 2. Materials and Methods

### 2.1. Materials

The CF T300B were provided by Toray industries, Inc. (Tokyo, Japan). The average value of their diameter is around 7 μm. The CF was firstly cleaned with acetone for 2 h, which was non-coated CF. The CNTs (multiwalled carbon nanotubes, model: TNMH1) with in 10–30 μm length and <8 nm diameter was supplied by Chengdu institute of organic chemistry, Chinese academy of sciences (Chengdu, China). The GnPs used in this study was from the Sixth element materials technology Co., Ltd. (Changzhou, China), in which the average surface area around and particle size were 206 m^2^/g and 7 μm (model: SE1430), respectively. The epoxy resin and curing agent were E51 (Hubei shuangfeng chemical Co. Ltd., Wuhan, China) and triethylene tetramine (TETA, Sinopharm chemical reagent Co., Ltd., Shanghai, China), respectively. They were used as received. The solvent of the sizing process was N, N-dimethylformamide (DMF, Xilong scientific Co., Ltd., Shantou, China). The DMF was used as received as well.

### 2.2. Preparation of GnPs/CNTs Hybrid Coated CF

**Figure 1:**
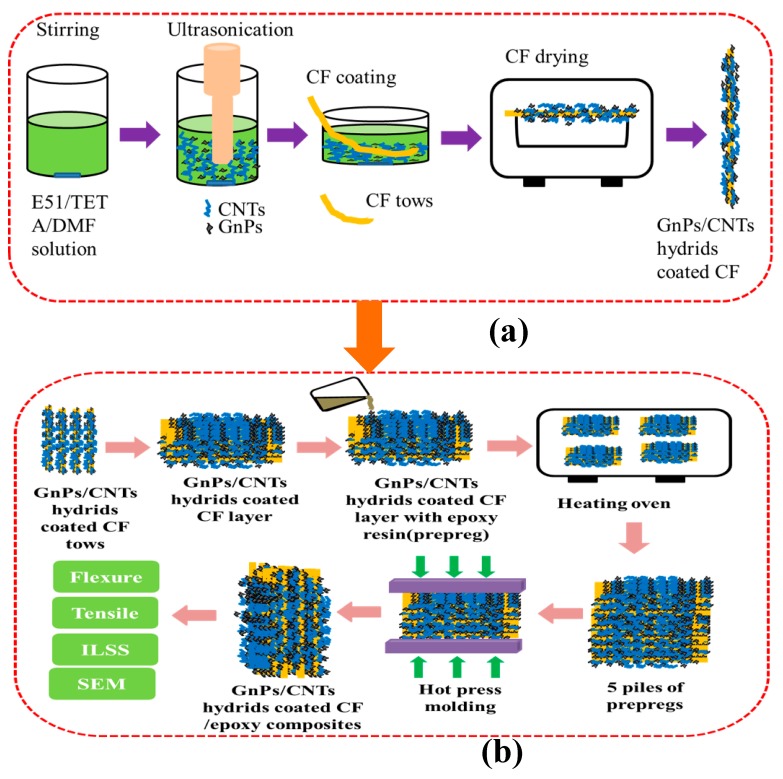
(**a**) the schematic of graphene nanoplates (GnPs)/carbon nanotubes (CNTs) hybrid coated carbon fiber (CF) and (**b**) the manufactured of the carbon fiber composites laminate.

A schematic illustration of the CF coated by GnPs/CNTs hybrid coating is shown in Figure 1a. Firstly, the mix of E51 and TETA (the weight ratio is 20:1) were dissolved in DMF at a concentration of 0.5 wt.% and stirred for 30 min. The mix of GnPs and CNTs were dispersed in the solution by ultrasonic with an output power of 200 W for 1h to obtain a stable sizing solution at a concentration of 0.5 wt.%. The weight ratio of GnPs to CNTs was 1:4. Then CF tows were immersed in the GnPs/CNTs hybrid sizing for 20 s and slowly pulled out. Subsequently, the CF tows were dried in a drying oven at 60 °C for 6 h. The coated CF were designated as GnPs/CNTs hybrid coated CF. Similar procedures were proceeded to coat either GnPs or CNTs on CF with a concentration of 0.5 wt.% GnPs or CNTs in the suspension. For epoxy coated CF, 0.5 wt.% E51 and TETA mixture (20:1 weight ratio) was dissolved in DMF, and CF were immersed into epoxy solution for 20 s. Then the coated CF were dried according to the same process used in GnPs/CNTs coated CF.

### 2.3. Fabrication of Carbon Fiber Reinforcement Epoxy Composites

#### 2.3.1. Impregnation of CF with Epoxy Resin (Prepreg)

A layer of CFs was neatly and tightly wound around on a template with release paper and the epoxy matrix was evenly permeated on it. The epoxy matrix was the mixture E51 and TETA, which the weight ratio was 10:1. Then the CFRP (pregreg) was placed in a vacuum oven and exhausted for 1 h at 35 °C.

#### 2.3.2. Hot Press Molding Composite Processing

Five layers of CFRP pregregs were laid up to a mold and placed on a hot press machine to prepare CFRP composites. The curing condition was 10 MPa for 2 h at 120 °C. The fiber volume fraction in the final composite laminates was about 60%. A schematic demonstration of the manufactured of the CFRP composites laminate is shown in Figure 1b.

### 2.4. Characterization

The surface of morphologies of the CF were observed by field-emission scanning electron microscopy (SEM, FEI Nova Nano SEM450, FEI, Hillsboro, OR, USA) with a 15 kV accelerating voltage. The fracture morphologies of the CFRP composites after flexural test was characterized by SEM with an accelerating voltage of 10 kV.

**Figure 2:**
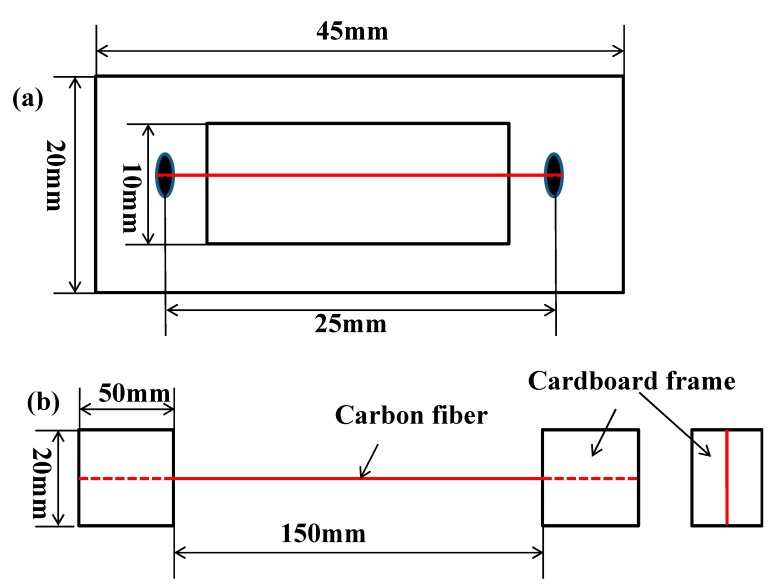
The schematic of (**a**) monofilament CF tensile specimen and (**b**) multifilament tensile specimen.

The monofilament tensile was measured according to GB/T 31290-2014 [42]. The monofilament CF specimen ends were attached to a cardboard square frame using an ailete 502 quick-drying adhesive, as shown in Figure 2a. It should ensure that the CF specimens were straight in the frame. The ends of the square frame were fixed in the chucks of the tester, and then frame was cut so that only the CF specimen was loaded during the test. A universal testing machine (WD-1, Shenzhen Suns Technology Stock Co., Ltd., Shenzhen, China) was used in the testing. The tension rate was set at 1 mm/min and the number of specimens tested under each condition was set at 30.

The multifilament tensile was tested according to GB/T 3362-2005 [43]. First, the E51 and TETA were dissolved in acetone, which the weight ratio of E51 to TETA to acetone is 10:1:15. A bundle of CF tow was immersed in the epoxy solution for 1min. Then the CF took out and dried at 120 °C for 1 h. The CF was kept straight in this drying process. The ends of CF were attached on the cardboard frame using adhesive, as shown in Figure 2b. The tension rate was set at 10 mm/min under the universal testing machine (WD-1) was used in the testing. More than six specimens were tested under each condition.

**Figure 3:**
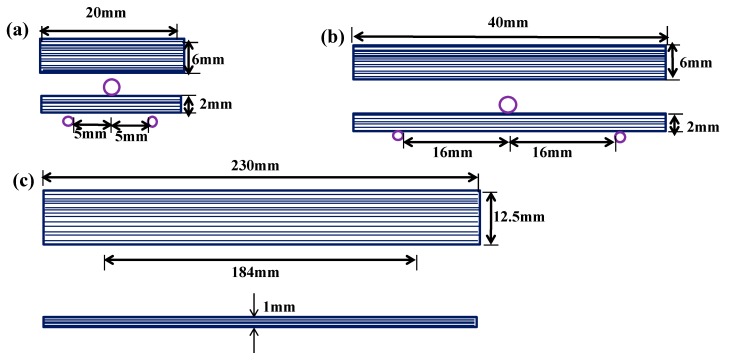
The schematic of (**a**) interlaminar shear strength (ILSS) test specimen, (**b**) flexural test specimen and (**c**) tensile test specimen.

The interfacial properties of CFRP composites were characterized with interlaminar shear strength (ILSS). The three-point short beam shear test was employed to measure the ILSS with the universal testing machine (WD-1), following GB/T 3357-1982 [44]. The schematic of ILSS is shown in Figure 3a. The span-to thickness ratio was 10 and the cross-head rate was 1 mm/min. The number of specimens tested under each condition was set at 10. 

The flexural test and tensile test were performed on the universal testing machine (WD-1), following the GB/T 1449-2005 [45] and GB/T 3354-2014 [46], respectively. The cross-head speed of the flexural test and tensile test were set at 10 mm/min and 1mm/min, respectively. The schematic of flexural test and tensile test are respectively shown in Figure 3b,c. At least five specimens were tested for each condition to obtain an average value. 

## 3. Results and Discussion

### 3.1. Morphology of GnPs/CNTs Hybrid Coated Carbon Fiber

**Figure 4:**
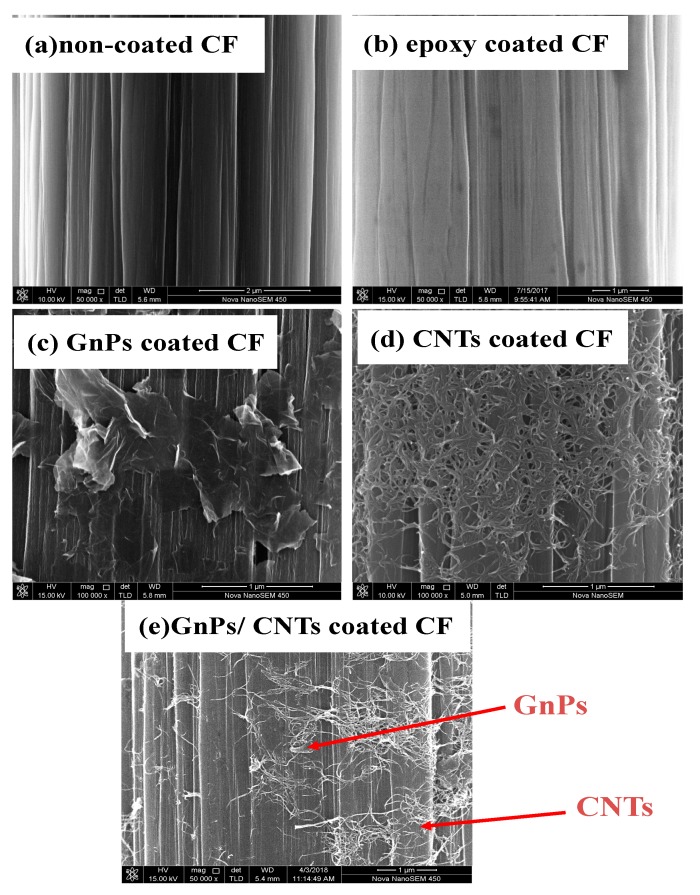
The surface morphology of CF: (**a**) non-coated CF, (**b**) epoxy coated CF, (**c**) GnPs coated CF, (**d**) CNTs coated CF and (**e**) GnPs/CNTs coated CF.

Figure 4 shows the surface morphologies of non-coated CF, epoxy coated CF, the GnPs coated CF, CNTs coated CF and the GnPs/CNTs hydrid coated CF. Relatively smooth surfaces were observed on non-coated CF (Figure 4a). The continuous grooves and ridges in the fiber axis were showed on non-coated CFs, which arose during the spinning of the PAN precursor. The morphology of epoxy coated CFs was similar to non-coated CF (Figure 4b). While the CF surface topographies were changed with the modification of carbon nanomaterials. The GnPs were successfully and uniformly distributed on CF surfaces at the same modification conditions (Figure 4c). Figure 4d displays that the CNTs were deposited on CF surfaces. Few CNTs aggregation were seen on CF surfaces. The GnPs/CNTs hydrid coating layer uniformly distributed on CF surfaces (Figure 4e). The surface contact points and interaction between CF and epoxy matrix would be enhanced when the GnPs and CNTs attached on CF surfaces, which is of benefit to increase the interfacial and mechanical properties of CFRP.

### 3.2. Monofilament and Multifilament Tensile Strength of GnPs/CNTs Hybrid Coated CF 

Monofilament and multifilament fiber tensile strength tests were conducted to assess the effect of the different coating on the CF tensile strength. Table 1 provides the monofilament and multifilament tensile strength of different coating CF. As shown in Table 1, after treatment with epoxy coating, the monofilament and multifilament tensile strength of CF increase to 3% and 4%, respectively, compared with non-coated CF, which may be due to some defects of CF surface healed by epoxy sizing. The monofilament and multifilament tensile strength of CF with GnPs coating enhance to 14% and 71%, respectively, compared with non-coated CF, which means the flaws of CF surface healed by GnPs coating. The monofilament and multifilament tensile strengths of CF with CNTs coating improve to 17 % and 72%, respectively, compared with non-coated CF, which means that the CNTs coating repairs the defect of CF surface. Several reasons can explain this improvement. First, a protective layer on the CF surface is generated by GnPs and CNTs, which effectively remedies CF surface defects, reduces stress concentration and inhibits crack growth. Second, GnPs and CNTs act as a bridge, generating a more efficient stress transfer. What is more, the high strength and modulus of graphene improves the tensile elongation of the fiber. After modifying with GnPs/CNTs hydrid coating, the monofilament and multifilament tensile strengths of CF increase significantly to 28% and 75%, respectively, compared with non-coated CF, which indicates that the GnPs/CNTs hydrid coating in CF surface has a better effect on healing defect of CF surface than either GnPs or CNTs.

### 3.3. Interfacial Property Testing of Carbon Fiber/Epoxy Laminated Composites

**Figure 5:**
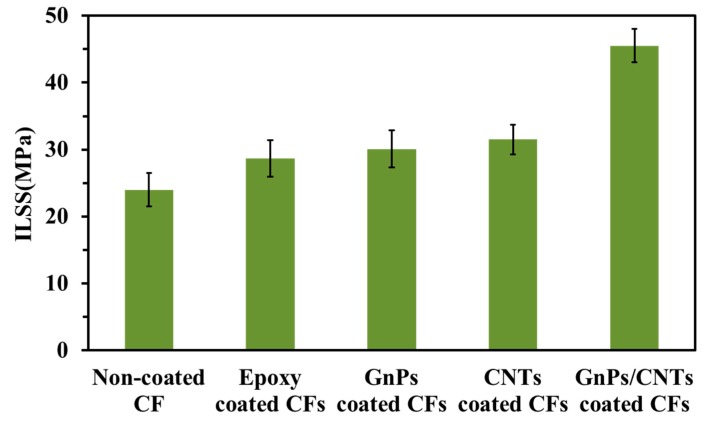
The ILSS of CF/EP composites.

The ILSS of CFRP composites were shown in Figure 5. The ILSS value of non-coated CFRP composites is 24 MPa. The ILSS of CFRP composites with epoxy coating slightly enhanced to 20% compared with non-coated CFRP composites, due to the increased of the surface wettability. It is shown that the ILSS of GnPs, CNTs and GnPs/CNTs hybrid coated CFRP composites is about 71%, 73% and 90% higher than that of non-coated CFRP composites, respectively. The result indicates that the interfacial properties of CFRP composites can be obviously improved by introducing GnPs, CNTs and GnPs/CNTs hybrid into the interphase of the CFRP composites. The GnPs/CNTs hybrid coating is the most effective in improving the ILSS, which clearly indicates the synergetic effect. 

**Figure 6:**
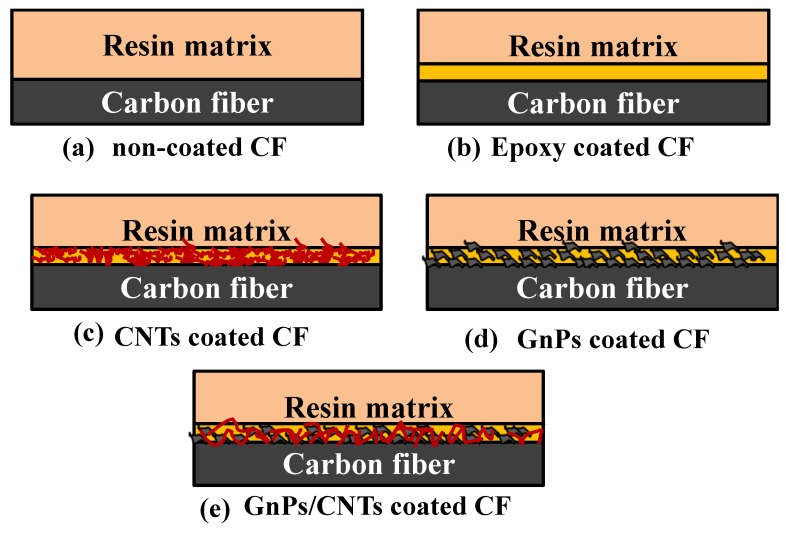
Schematic of the interphase of the composites: (**a**) non-coated CF, (**b**) epoxy coated CF, (**c**) GnPs coated CF, (**d**) CNTs coated CF and (**e**) GnPs/CNTs coated CF.

This synergetic improvement of GnPs/CNTs hybrid coated CFRP composites mechanisms can be summarized as three aspects. (1) Flexible CNTs can infiltrate between the GnPs, which prevent face-to-face aggregation of multigraphene platelets. The GnPs also can permeate between the CNTs, forming complementary structures that are able to interact and restrain restacking as a result of Van der Waals attraction [38]. Therefore, it is supposed that the GnPs/CNTs hybrid coating is easy to compose a 3D hybrid carbon nanomaterials structure. This 3D hybrid network results in a large surface area, improving the contact surface area and interlock at the CF–matrix interface. So, the interfacial adhesion is improved. (2) The CNTs can play as bridge in the 3D hybrid structure, that can be tangled with matrix chain and give rise to better interaction between CF and matrix [39]. (3) GnPs/CNTs on the CF surface increase the toughness of the EP nearby the interface by pulling out and causing plastic deformation of the EP, which is beneficial to interfacial stress transfer and resistance to strain, so that the shear failure process of interface can dissipate more energy, thus, effectively enhancing the ILSS of GnPs/CNTs hybrid coated CFRP composites [22]. Figure 6 exhibits the exact structure differences of the interphase region of CFRP composites.

### 3.4. Flexural and Tensile Strength of GnPs/CNTs Hybrid Coated CFRP Composites

To examine the effect of different coatings on the mechanical properties of composites, the flexural and tensile strength of CFRP composited were measured and the result are shown in Table 2. As can be seen, the flexural and tensile strength for the non-coated CFRP composites exhibited 210 MPa and 200 MPa, respectively. The epoxy coated CFRP composites exhibited that the flexural and tensile strength were 220 MPa and 250 MPa, respectively. Epoxy increases the wettability of CF surface and enhances the mechanical properties of CFRP composites. The flexural strength of GnPs and CNTs coated CFRP composites were 300 MPa and 300 MPa, which both have an increase of 43%. Furthermore, tensile strength of GnPs and CNTs coated CFRP composites were 300 MPa and 300 MPa, which both have an 50% improvement. Furthermore, after GnPs/CNTs hybrid deposition on the CF surface, the flexural strength of CFRP composites rose to 320 MPa, an increase of 52%, and the tensile strength of CFRP composites improve to 340 MPa, an improvement of 70%. The result indicates that the GnPs/CNTs hybrid coated CFRP composites exhibit the highest values of flexural and tensile strength for all cases, which demonstrates the synergetic effect of GnPs and CNTs in hybrid coating.

### 3.5. Fractured Surfaces Analysis of CFRP Composites

**Figure 7:**
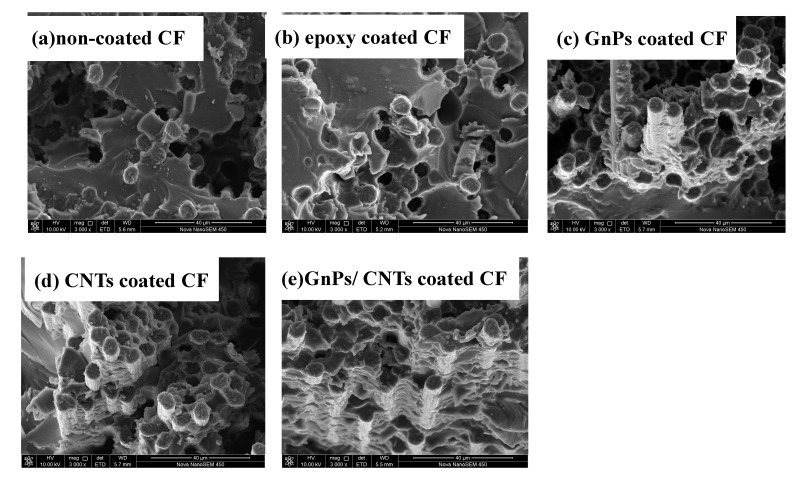
SEM images of fractured surface of CF/EP composites: (**a**) non-coated CF, (**b**) epoxy coated CF, (**c**) GnPs coated CF, (**d**) CNTs coated CF and (**e**) GnPs/CNTs coated CF.

To further study the improvement mechanism and interfacial behavior of the CFRP composites, the fracture morphology of CFRP composites after flexural strength test were studied and are displayed in Figure 7. For the non-coated CFRP, large amounts of CF were pulled out of the matrix and the pullout CF surface is almost clean without matrix (Figure 7a), which indicated weak interfacial adhesion between non-coated CF and matrix. For the epoxy coated CFRP composites, the number of CF pulled out were less than non-coated CFs (Figure 7b), which indicated that the epoxy improved the interfacial properties of CFRP composites. Whereas, for the GnPs coated CFRP and the CNTs coated CFRP composites, many resin matrixes remain on the surface of CF and few CF is pulled out. It indicates that the interfacial adhesion between CF and epoxy matrix increases, as shown in Figure 7c,d. The fractured surface was not distinct difference between GnPs coated CFRP composites and the CNTs coated CFRP composites. Meanwhile, the flexural strength of the GnPs coated CFRP composites and the CNTs coated CFRP composites were notably close. Furthermore, in the case of the GnPs/CNTs hybrid coated CFRP composites (Figure 7e), the holes were seldom found, and the amount of matrix attached on the pullout CF surface was more than others CFRP composites, which demonstrates that the highest interfacial adhesion in the GnPs/CNTs hybrid coated CFRP composites. The surface wettability and roughness of the CF were increased after CF were modified by carbon nano-particles (GnPs, CNTs and GnPs/CNTs hybrid), the interaction area between CF and epoxy matrix were increased and the stress concentration were reduced when the carbon nanomaterials in the composites interphase region which effectively increased the interfacial and mechanical properties of CFRP composites.

## 4. Conclusions

GnPs/CNTs hybrid coating have been deposited on CF surface by a fiber sizing method and the CFRP composites with uniformly dispersed GnPs/CNTs hybrid were manufactured. 2D GnPs and 1D CNTs were combined to form a 3D network structure which exhibited a great synergetic effect of the hybrid coating in increasing the interfacial and mechanical properties of CFRP composites. As compared, the individual GnPs or CNTs coating alone has been deposited on CF surface. The ILSS, flexural and tensile strength of GnPs/CNTs hybrid coated CFRP composites were the highest in all the samples, which were 90%, 52% and 70%, respectively, improvement compared with non-coated CFRP composites. The GnPs/CNTs hybrid as high-performance multifunctional modifier offers great advantages for various applications in the future. Consequently, owing to easy process of GnPs/CNTs hybrid coating on CF, the fiber sizing method is a general promising approach to obtain hybrid nanoparticles coated CFRP composites with high interfacial and mechanical properties.

## Figures and Tables

**Table 1:** The monofilament and multifilament tensile strength of carbon fiber reinforcement epoxy composites (CFRP) composites.

Samples	Monofilament Tensile Strength (GPa)	Relative Increase (%)	Multifilament Tensile Strength (MPa)	Relative Increase (%)
Non-coated CF	2.9 ± 0.2	-	1060 ± 90	-
Epoxy coated CF	3.0 ± 0.4	3	1100 ± 70	4
GnPs coated CF	3.3 ± 0.1	14	1810 ± 70	71
CNTs coated CF	3.4 ± 0.1	17	1820 ± 80	72
GnPs/CNTs coated CF	3.7 ± 0.1	28	1850 ± 80	75

**Table 2:** The flexural strength and tensile strength of CF/EP composites.

Samples	Flexural Strength (MPa)	Relative Increase (%)	Tensile Strength (MPa)	Relative Increase (%)
Non-coated CF	210 ± 30	-	200 ± 30	-
Epoxy coated CF	220 ± 30	5	250 ± 30	25
GnPs coated CF	300 ± 30	43	300 ± 30	50
CNTs coated CF	300 ± 40	43	300 ± 30	50
GnPs/CNTs coated CF	320 ± 30	52	340 ± 40	70

## References

[1] Islam M.S., Deng Y., Tong L., Faisal S.N., Roy A.K., Minett A., Gomes V.G. (2016). Grafting carbon nanotubes directly onto carbon fibers for superior mechanical stability: Towards next generation aerospace composites and energy storage applications. Carbon.

[2] Guermazi N., Ben Tarjem A., Ksouri I., Ayedi H.F. (2016). On the durability of FRP composites for aircraft structures in hygrothermal conditioning. Compos. Part B Eng..

[3] Kim K.W., Kim D.K., Kim B.S., An K.-H., Park S.-J., Rhee K.Y., Kim B.-J. (2017). Cure behaviors and mechanical properties of carbon fiber-reinforced nylon6/epoxy blended matrix composites. Compos. Part B Eng..

[4] Sun T., Li M., Zhou S., Liang M., Chen Y., Zou H., Liang S.Z.M. (2020). Multi-scale structure construction of carbon fiber surface by electrophoretic deposition and electropolymerization to enhance the interfacial strength of epoxy resin composites. Appl. Surf. Sci..

[5] Pathak A.K., Borah M., Gupta A., Yokozeki T., Dhakate S.R. (2016). Improved mechanical properties of carbon fiber/graphene oxide-epoxy hybrid composites. Compos. Sci. Technol..

[6] Drzal L.T., Madhukar M. (1993). Fibre-matrix adhesion and its relationship to composite mechanical properties. J. Mater. Sci..

[7] Chen J., Wang K., Zhao Y. (2018). Enhanced interfacial interactions of carbon fiber reinforced PEEK composites by regulating PEI and graphene oxide complex sizing at the interface. Compos. Sci. Technol..

[8] Mamalis D., Flanagan T., O’Brádaigh C.M. (2018). Effect of fibre straightness and sizing in carbon fibre reinforced powder epoxy composites. Compos. Part A Appl. Sci. Manuf..

[9] Xiong L., Zhan F., Liang H.B., Chen L., Lan D. (2018). Chemical grafting of nano-TiO2 onto carbon fibervia thiol-ene click chemistry and its effect on the interfacial and mechanical properties of carbonfiber/epoxy composites. J. Mater. Sci..

[10] Sun J., Zhao F., Yao Y., Jin Z., Liu X., Huang Y. (2017). High efficient and continuous surface modification of carbon fibers with improved tensile strength and interfacial adhesion. Appl. Surf. Sci..

[11] Vedrtnam A. (2019). Novel method for improving fatigue behavior of carbon fiber reinforced epoxy composite. Compos. Part B Eng..

[12] Han W., Zhang H.-P., Tavakoli J., Campbell J., Tang Y. (2018). Polydopamine as sizing on carbon fiber surfaces for enhancement of epoxy laminated composites. Compos. Part A Appl. Sci. Manuf..

[13] Jiang J., Yao X., Xu C., Su Y., Zhou L., Deng C. (2017). Influence of electrochemical oxidation of carbon fiber on the mechanical properties of carbon fiber/graphene oxide/epoxy composites. Compos. Part A Appl. Sci. Manuf..

[14] Wang C., Li J., Yu J., Sun S., Li X., Xie F., Jiang B., Wu G., Yu F., Huang Y. (2017). Grafting of size-controlled graphene oxide sheets onto carbon fiber for reinforcement of carbon fiber/epoxy composite interfacial strength. Compos. Part A Appl. Sci. Manuf..

[15] Ma K., Wang B., Chen P., Zhou X. (2011). Plasma treatment of carbon fibers: Non-equilibrium dynamic adsorption and its effect on the mechanical properties of RTM fabricated composites. Appl. Surf. Sci..

[16] Yuan J.-M., Fan Z.-F., Yang Q.-C., Li W., Wu Z.-J. (2018). Surface modification of carbon fibers by microwave etching for epoxy resin composite. Compos. Sci. Technol..

[17] Tsai S.-N., Carolan D., Sprenger S., Taylor A.C. (2019). Fracture and fatigue behaviour of carbon fibre composites with nanoparticle-sized fibres. Compos. Struct..

[18] Blackketter D.M., Upadhyaya D., King T.R., King J.A. (1993). Evaluation of fiber surfaces treatment and sizing on the shear and transverse tensile strengths of carbon fiber-reinforced thermoset and thermoplastic matrix composites. Polym. Compos..

[19] Malekimoghadam R., Icardi U. (2019). Prediction of mechanical properties of carbon nanotube‒carbon fiber reinforced hybrid composites using multi-scale finite element modelling. Compos. Part B Eng..

[20] Matveeva A.Y., Lomov S.V., Gorbatikh L. (2019). Debonding at the fiber/matrix interface in carbon nanotube reinforced T composites: Modelling investigation. Comput. Mater. Sci..

[21] Qin W., Vautard F., Drzal L.T., Yu J. (2014). Modifying the carbon fiber-epoxy matrix interphase with graphite nanoplatelets. Polym. Compos..

[22] Xiao C., Tan Y., Wang X., Gao L., Wang L., Qi Z. (2018). Study on interfacial and mechanical improvement of carbon fiber/epoxy composites by depositing multi-walled carbon nanotubes on fibers. Chem. Phys. Lett..

[23] Kwon Y.J., Kim Y., Jeon H., Cho S., Lee W., Lee J.U. (2017). Graphene/carbon nanotube hybrid as a multi-functional interfacial reinforcement for carbon fiber-reinforced composites. Compos. Part B Eng..

[24] Ismail K., Sultan M.T.H., Shah A., Jawaid M., Safri S. (2019). Low velocity impact and compression after impact properties of hybrid bio-composites modified with multi-walled carbon nanotubes. Compos. Part B Eng..

[25] Liang X., Cheng Q. (2018). Synergistic reinforcing effect from graphene and carbon nanotubes. Compos. Commun..

[26] Li M., Gu Y., Liu Y., Li Y., Zhang Z. (2013). Interfacial improvement of carbon fiber/epoxy composites using a simple process for depositing commercially functionalized carbon nanotubes on the fibers. Carbon.

[27] Yao H., Sui X., Zhao Z., Xu Z., Chen L., Deng H., Liu Y., Qian X. (2015). Optimization of interfacial microstructure and mechanical properties of carbon fiber/epoxy composites via carbon nanotube sizing. Appl. Surf. Sci..

[28] Li Y., Umer R., Isakovic A., Samad Y.A., Zheng L., Liao K. (2013). Synergistic toughening of epoxy with carbon nanotubes and graphene oxide for improved long-term performance. RSC Adv..

[29] Su Y.-N., Zhang S., Zhang X.-H., Zhao Z.-B., Chen C.-M., Jing D.-Q. (2017). Preparation and properties of graphene/carbon fiber/poly(ether ether ketone) composites. Carbon.

[30] Naveen J., Jawaid M., Zainudin E.S., Sultan M.T.H., Yahaya R. (2019). Improved Mechanical and Moisture-Resistant Properties of Woven Hybrid Epoxy Composites by Graphene Nanoplatelets (GNP). Materials.

[31] Qin W., Vautard F., Drzal L.T., Yu J. (2015). Mechanical and electrical properties of carbon fiber composites with incorporation of graphene nanoplatelets at the fiber–matrix interphase. Compos. Part B Eng..

[32] Wang P.-N., Hsieh T.-H., Chiang C.-L., Shen M.-Y. (2015). Synergetic Effects of Mechanical Properties on Graphene Nanoplatelet and Multiwalled Carbon Nanotube Hybrids Reinforced Epoxy/Carbon Fiber Composites. J. Nanomater..

[33] Loos M., Yang J., Feke D.L., Manas-Zloczower I. (2012). Effect of block-copolymer dispersants on properties of carbon nanotube/epoxy systems. Compos. Sci. Technol..

[34] Qi X.-Y., Yan N., Jiang Z., Cao Y.-K., Yu Z.-Z., Yavari F., Koratkar N. (2011). Enhanced Electrical Conductivity in Polystyrene Nanocomposites at Ultra-Low Graphene Content. ACS Appl. Mater. Interfaces.

[35] Hung M.-T., Choi O., Ju Y.S., Hahn H.T. (2006). Heat conduction in graphite-nanoplatelet-reinforced polymer nanocomposites. Appl. Phys. Lett..

[36] Worsley K.A., Kalinina I., Bekyarova E., Haddon R. (2009). Functionalization and Dissolution of Nitric Acid Treated Single-Walled Carbon Nanotubes. J. Am. Chem. Soc..

[37] Ladani R., Bhasin M., Wu S., Ravindran A.R., Ghorbani K., Zhang J., Kinloch A.J., Mouritz A.P., Wang C.H. (2018). Fracture and fatigue behaviour of epoxy nanocomposites containing 1-D and 2-D nanoscale carbon fillers. Eng. Fract. Mech..

[38] Yue L., Pircheraghi G., Monemian S., Manas-Zloczower I. (2014). Epoxy composites with carbon nanotubes and graphene nanoplatelets—Dispersion and synergy effects. Carbon.

[39] Yang S.-Y., Lin W.-N., Huang Y.-L., Tien H.-W., Wang J.-Y., Ma C.-C.M., Li S.-M., Wang Y.-S. (2011). Synergetic effects of graphene platelets and carbon nanotubes on the mechanical and thermal properties of epoxy composites. Carbon.

[40] Yu A., Ramesh P., Sun X., Bekyarova E., Itkis M.E., Haddon R. (2008). Enhanced Thermal Conductivity in a Hybrid Graphite Nanoplatelet—Carbon Nanotube Filler for Epoxy Composites. Adv. Mater..

[41] Xiong X.-Q., Bao Y.-L., Liu H., Zhu Q., Lu R., Miyakoshi T. (2019). Study on mechanical and electrical properties of cellulose nanofibrils/graphene-modified natural rubber. Mater. Chem. Phys..

[42] (2014). Carbon Fiber- Determination of the Tensile Properties of Single-Filament Specimens.

[43] (2005). Test Methods for Tensile Properties of Carbon Fiber Multifilament.

[44] (1982). Test Method for Interlaminar Shear Strength of Unidirectional Fiber-Reinforced Plastics.

[45] (2005). Fiber-Reinforced Plastic Composites-Determination of Flexural Properties.

[46] (2014). Test Method for Tensile Properties of Orientation Fiber Reinforced Polymer Matrix Composites Materials.

